# Targeting the PANoptosis signaling pathway for myocardial protection: therapeutic potential of Xian Ling Gu Bao capsule

**DOI:** 10.3389/fphar.2024.1391511

**Published:** 2024-05-10

**Authors:** Xiaoyu Wu, Jiajia Wei, Wenfang Zhang, Yang Yi, Tingting Wang, Qihai Gong, Xin Liu, Haibo Li, Jianmei Gao

**Affiliations:** ^1^ Key Laboratory of Basic Pharmacology of Ministry of Education and Joint International Research Laboratory of Ethnomedicine of Ministry of Education, Zunyi Medical University, Zunyi, China; ^2^ Department of Pharmacology, Key Laboratory of Basic Pharmacology of Guizhou Province and School of Pharmacy, Zunyi Medical University, Zunyi, China; ^3^ School of Traditional Chinese Medicine, Liaoning University of Traditional Chinese Medicine Shenyang, Shenyang, China

**Keywords:** myocardial infarction, Xian Ling Gu Bao capsule, oxidative stress, inflammation, PANoptosis

## Abstract

**Introduction:** Myocardial infarction (MI), the most prevalent ischemic heart disease, constitutes a primary cause of global cardiovascular disease with incidence and mortality. The pathogenesis of MI is exceedingly intricate, with PANoptosis playing a pivotal role in its pathological process. Xian Ling Gu Bao capsule (XLGB) contains various active components, including flavonoids, terpenes, and phenylpropanoids, and exhibits a wide range of pharmacological activities. However, it remains unclear whether XLGB can protect the myocardium from damage after MI. This study aimed to investigate the impact of XLGB on isoprenaline (ISO)-induced MI in mice and its potential mechanisms.

**Methods:** This study assessed the protective effects of XLGB against ISO-induced MI through techniques such as echocardiography, HE staining, Masson staining, and enzyme-linked immunosorbent assay (ELISA). Furthermore, the potential mechanisms of XLGB's protective effects on MI were explored using bioinformatics, molecular docking, and molecular dynamics simulations. These mechanisms were further validated through immunofluorescence staining and Western blotting.

**Results:** The results demonstrated that various doses of XLGB exhibited a significant reduction in myocardial injury induced by myocardial infarction. Intriguingly, higher dosages of XLGB displayed superior therapeutic efficacy compared to the positive control metoprolol. This protective effect is primarily achieved through the inhibition of oxidative stress and the inflammatory processes. Furthermore, we have elucidated that XLGB protected the myocardium from MI-induced damage by suppressing PANoptosis, with a critical role played by the NLRP3/Caspase3/RIP1 signaling pathway. Of particular note, the primary compounds of XLGB were found to directly interact with NLRP3/Caspase3/RIP1, a discovery further validated through molecular docking and molecular dynamics simulations. This suggests that NLRP3/Caspase3/RIP1 may be a therapeutic target for XLGB-induced myocardial protection.

**Conclusion:** In summary, our findings reveal a novel property of XLGB: reverses myocardial damage following MI by inhibiting the NLRP3/Caspase3/RIP1-mediated PANoptosis pathway.

## 1 Introduction

Myocardial infarction (MI) is the most common form of ischemic heart disease and a leading cause of global morbidity and mortality. It is characterized by the obstruction of coronary artery blood flow, depriving myocardial cells of oxygen and nutrients, ultimately leading to myocardial cell death (Ramachandra et al., 2020). Regrettably, although current therapies (e.g., percutaneous coronary intervention, coronary artery bypass grafting) could ameliorate mortality rate of MI patients, complications occur involving myocardial ischemia-reperfusion injury, hemorrhage and coronary restenosis limit their clinical application. Therefore, it is necessary to develop more innovative and efficient avenues to restore myocardial function and prevent heart failure progress.

Emerging evidence indicates that oxidative stress (OS) and inflammation are considered as major triggers of MI (Heusch, 2020). During ischemia, myocardial cells cannot receive sufficient oxygen, resulting in an imbalance in redox reactions and the generation of increased levels of reactive oxygen species (ROS) (Wu et al., 2022). This results in the extensive release of immune cells, inflammatory mediators, and cytokines, causing more severe damage to myocardial tissue (Liu X. et al., 2023). The inflammatory response can persist for several days to weeks, during which cell infiltration and fibrosis occur, leading to adverse physiological responses. These reactions not only hinder myocardial regeneration but also increase the risk of cardiac remodeling and reshaping after MI, thus becoming a critical factor in post-MI complications. Therefore, controlling OS and cellular inflammation is considered crucial in the treatment and prevention of MI. Most recently, OS and inflammatory stimuli can activate PANoptosis, which is originally defined as “combines the main features of pyroptosis, apoptosis, and necroptosis” (Tong et al., 2023). Thus, reducing ROS overproduction and inflammation by hindering PANoptosis can be a promising tactics to overcome MI.

XLGB, a well-known traditional Chinese medicine prescription, is known for its effects in nourishing yin and blood, promoting blood circulation, resolving blood stasis, regulating the immune system, and enhancing bone density (He et al., 2022). Numerous studies have found that XLGB has a variety of active ingredients, including flavonoids, terpenes, and phenylpropanoids, etc., (Guan et al., 2011; Tang et al., 2020). Clinically, XLGB is primarily used as an adjuvant treatment for orthopedic conditions such as osteoporosis, arthritis, and rheumatism. Studies have reported that the main botanical drugs of XLGB, *Epimedium brevicornu* Maxim. and its active metabolites, exhibit significant therapeutic effects on MI(Li Y. et al., 2022; Zeng et al., 2022). Additionally, *Dipsacus asper* Wall. ex DC. (Hassan et al., 2020), *Salvia miltiorrhiza* Bunge (Zhang Y. et al., 2022), *Anemarrhena asphodeloides* Bunge (Zhang et al., 2020), and other ingredients have potent anti-inflammatory and antioxidant activities. However, whether XLGB can rescue MI remains still undefined.

Thus, the aim of the present study was designed to probe whether XLGB could alleviate isoprenaline (ISO)-induced MI in mice. We also addressed the potential protective mechanism of XLGB on MI. The ultimate goal of this study is to offer a pharmacological foundation for expanding the clinical applications of XLGB.

## 2 Materials and methods

### 2.1 Materials

XLGB capsule consists of six commonly botanical drugs: *Epimedium brevicornu* Maxim. [Berberidaceae; *Epimedium brevicornu* leave] (70%), *Dipsacus asper* Wall. ex DC. [Caprifoliaceae; *Dipsacus asper* radix] (10%), *Salvia miltiorrhiza* Bunge [Lamiaceae; *Salviae miltiorrhizae* radix et rhizome] (5%), *A. asphodeloides* Bunge [Asparagaceae; *A. asphodeloides* rhizome] (5%), *Cullen corylifolium* (L.) Medik. [Fabaceae; *Cullen corylifolium* seed] (5%) and *Rehmannia glutinosa* (Gaertn.) DC. [Orobanchaceae; *R. glutinosa* radix] (5%) (Chen et al., 2019). XLGB was purchased from China National Pharmaceutical Group Corporation Tongjitang Pharmaceutical Co., Ltd. (Guizhou, China, National Medical Product Approval No. Z20025337). Metoprolol (Met) Tartrate Tablets was obtained from AstraZeneca (Guizhou, China; lot number:1611A74). ISO was procured from Southwest Pharmaceutical Co., Ltd. (National Medical Product Approval No. H50020020, China). The masson staining kit (G1340) and BCA protein assay kit (PC0020) was obtained from Solarbio (Beijing, China). The terminal deoxynucleotidyl transferase dUTP nick end labeling (TUNEL) apoptosis detection kit (Cat No. C1088), antifade mounting medium with DAPI (P0131), immunostaining fixation solution (P0098), and immunostaining blocking solution (P0102) were sourced from Beyotime (Shanghai, China). Sodium citrate was purchased from Wuhan Servicebio Technology CO., LTD (G1201). Enzyme-linked immunosorbent assay (ELISA) kits for creatine kinase isoenzyme (CK-MB, RJ17221), cardiac troponin I (cTn-I, RJ23466), cardiac troponin T (cTnT, RJ23465), Myoglobin (MYO, RJ17218), aspartate aminotransferase (AST, RJ17150), interleukin-1β (IL-1β, RJ15465), interleukin-1α (IL-1α, RJ16918), tumor necrosis factor-α (TNF-α, RJ16622), interleukin-6 (IL-6, RJ15478), malondialdehyde (MDA, RJ15503), reactive oxygen species (ROS, RJ15780), catalase (CAT, RJ15737), superoxide dismutase (SOD, RJ16691), glutathione (GSH, RJ17183), and glutathione peroxidase (GSH-Px, RJ25745) were purchased from Shanghai Renjie Bioengineering Institute. Antibodies against NLRP3 (ab263899), Caspase3 (ab184787), Cleaved-caspase3 (ab214430), RIP1 (ab300617), RIP3 (ab286151), p-RIP3 (ab195117), MLKL (ab243142), p-MLKL (ab196436), and GAPDH (ab8245) were obtained from Abcam (Cambridge, UK), while N-GSDMD (20770-1-AP), Caspase1 (22915-1-AP), and p-RIP1 (66854-1-Ig), CoraLite 488 (SA00013-2), and CoraLite 594 (SA00013-4) antibodies were sourced from Proteintech (Wuhan, China), and Cleaved-caspase1 (#AF4005) were purchased from Affinity Biosciences.

### 2.2 Animal model and drug treatment

C57BL/6J mice (male, 10–12 weeks, 24–28 g) were procured from Hunan SJA Laboratory Animal Co., Ltd (Certificate number: SCXK 2019-0004, Hunan, China). All experimental animals were housed in the SPF-grade animal facility. The mice had *ad libitum* access to water and food, and the room temperature was maintained at approximately 25°C ± 2°C with a humidity level of (50 ± 5) %. The animals were subjected to a 12 h light/12 h dark cycle. Animal experiments strictly adhered to the regulations of the Ethics Committee for Animal Experiments at Zunyi Medical University (ZMU21-2203-538).

After 1 week of acclimatization, the animals were randomly divided into six groups (n = 20/group): control group, XLGB-H group (1,000 mg/kg/day), ISO group, ISO + XLGB-L group (500 mg/kg/day), ISO + XLGB-H group (1,000 mg/kg/day) and ISO + Met group (10 mg/kg/day). Subcutaneous injection of ISO, a synthetic catecholamine and β-adrenergic agonist, is commonly used to induce myocardial ischemia in animals (Saqib et al., 2022). According to the recommended human dose of XLGB is 3 g/day in clinic and calculated based on the body surface area conversion relationship, drug treated mice were gavage of 500 or 1,000 mg/kg/day XLGB for 7 days. Two hours after the ISO (100 mg/kg/day) subcutaneous injection, XLGB and Met were administered by gavage. Mice in control group and ISO group were given volume-matched saline.

### 2.3 Echocardiography

Following previously established protocols (Li Y. et al., 2022), mice were anesthetized with 3% (v/v) isoflurane, and their cardiac function was assessed using the small animal ultrasound system Vevo 2100 (VisualSonics Corporation, Canada). The evaluation parameters included ejection fraction (EF) and fractional shortening (FS) to assess heart function.

### 2.4 Heart weight index and histopathological analysis

After blood collection from the eyeball, mice were euthanized using cervical dislocation. Briefly, the hearts were rapidly excised and the heart weight-to-body weight ratio (HW/BW) was then calculated. Subsequently, the hearts were fixed in 4% tissue cell fixation solution for 24 h. Tissue sections (4 μm) were cut using a Leica RM-2245 rotary microtome and placed on polylysine-coated slides after dehydrated and embedded in paraffin. Routine hematoxylin and eosin (H&E) staining was performed using a staining machine (Leica Autostainer XL, ST5010). Digital scans (×40) of these slides were obtained using the Teksqray SQS-1000 slide scanning imaging system, and the images were visualized using ImageViewer (DPVIEW V2.0) software.

### 2.5 Masson staining

Masson staining was used to assess the degree of myocardial fibrosis. In brief, tissue sections (4 μm) were deparaffinized using a staining machine (Leica Autostainer XL, ST5010). Subsequently, Masson’s trichrome staining (G1340, Solarbio) was performed on tissue sections following the manufacturer’s instructions. Digital scans (×4) of these stained sections were acquired using the Teksqray SQS-1000 slide scanning imaging system. These images were visualized using ImageViewer (DPVIEW V2.0) software, and the percentage of collagen fibers in the myocardial tissue was calculated using ImageJ software (https://ij.imjoy.io/).

### 2.6 Identification of major chemical ingredients of XLGB

The ultra performance liquid chromatography-quadrupole/electrostatic field orbitrap high resolution mass spectrometry (UPLC-QE-Orbitrap-HRMS) method was utilized to identify the chemical ingredients in XLGB and to establish the quality control of XLGB. Briefly, XLGB samples were taken and ground evenly using liquid nitrogen. Approximately 100 mg of the sample was weighed into a 1.5 mL centrifuge tube. Then, 1 mL of methanol-water solution (V:V = 3:1, containing mixed internal standards at 4 μg/mL) was added to the tube. The mixture was vortexed for 1 min, and steel beads were added. After pre-cooling at −40°C for 2 min in a refrigerator, the sample was ground using a grinder (60HZ, 2 min). The sample was then subjected to ultrasonic extraction in an ice water bath for 60 min, followed by settling at −40°C for 30 min. After centrifugation for 10 min (12,000 rpm, 4°C), the entire supernatant was passed through a 0.22 μm organic phase filter membrane. It was left to stand overnight at 4°C and then centrifuged again for 10 min (12,000 rpm, 4°C). The supernatant was diluted 10 times with a methanol-water solution (V:V = 3:1, containing mixed internal standards at 4 μg/mL). The entire supernatant was passed through a 0.22 μm organic phase filter membrane and loaded into an inner-lined LC-MS autosampler vial for analysis.

The chromatographic column used was a Waters ACQUITY UPLC HSS T3 (100 mm × 2.1 mm, 1.8 μm). The column temperature was set at 45°C. The flow rate was 0.35 mL/min, and the injection volume was 5 μL. A gradient elution was applied using a mobile phase consisting of 0.1% formic acid aqueous solution and acetonitrile. PDA scanning was conducted in the range of 210–400 nm. Negative and positive ion modes were employed, and full scanning was performed in the m/z range of 100–1,200. For the scanning analysis, the parameter settings were optimized as follows: spray voltage of 3.0 kV in negative ion mode and 3.8 kV in positive ion mode, capillary temperature of 320°C, sheath gas flow rate of 35 L/min, and auxiliary gas flow rate of 8 L/min. The collision energy for secondary mass spectra was increased in steps of 10, 20, and 40 eV. Scan modes included Full MS/dd-MS2, with a resolution of 70,000 in Full MS mode and 17,500 in dd-MS2 mode. The original data were processed using metabolomics software Progenesis QI v3.0 (Nonlinear Dynamics, Newcastle, UK), which included baseline filtering, peak recognition, integration, retention time correction, peak alignment, and normalization.

### 2.7 Potential therapeutic targets of XLGB against MI

Oral bioavailability (OB) is one of the most commonly used parameters for screening candidate ingredients with further pharmaceutical potential (Liu et al., 2023b). SMILES number of chemical ingredients (Mean ratio of peak area ≥0.1%) identified by UPLC-QE-Orbitrap-HRMS was input into the ADMETLab 2.0 platform, and ingredients meeting OB > 20% were screened as potential active ingredients (Xiong et al., 2021). Subsequently, target prediction for these ingredients was carried out using the SWISS TargetPrediction database (http://swisstargetprediction.ch/) to predict active ingredient target, set up the species for "*Homo sapiens*”, screening of aim-listed Probability >0 targets, delete duplicates, as potential drug targets of XLGB (Daina et al., 2019). In addition, the Encyclopedia of Traditional Chinese Medicine version 2.0 (ETCM v2.0, http://www.tcmip.cn/ETCM2/front/#/) and literature were used to retrieve candidate target genes of 6 Chinese botanical drugs respectively (Zhang et al., 2023). We predicted potential targets for ingredients using MedChem Studio (version 3.0), which is an efficient drug similarity search tool to identify known drugs with high structural similarity (Tanimoto score >0.8) to ingredients (Zhang Q. et al., 2022). The potential targets of XLGB were obtained by merging and removing duplicates from the targets corresponding to the ingredients identified by UPLC-QE-Orbitrap-HRMS and the targets obtained from, ETCM v2.0.

On the other hand, we conducted a search for gene expression profile data related to MI in the Gene Expression Omnibus (GEO) database (https://www.ncbi.nlm.nih.gov/geo/). We set the “Organism” parameter to “*H. sapiens*” to identify relevant gene chip datasets (GSE60993, GSE61144, GSE29532) (Silbiger et al., 2013; Park et al., 2015). The data retrieved from these gene chips were uniformly log-transformed using the HOME for Researchers platform (https://www.home-for-researchers.com/static/index.html#/). Subsequently, we used the limma package in the R software for batch correction and differentially expressed genes (DEGs) screening foldchange >1.5 and q-value <0.05. The PCA plot was drawn to illustrate the samples before and after batch effect. In addition, we used GeneCards (https://www.genecards.org/) MalaCards (https://www.malacards.org/pages/info/), DisGeNET (https://www.disgenet.org/) and Therapeutic target database (TTD, https://idrblab.org/ttd/) to obtain targets related to MI (Rappaport et al., 2017; Piñero et al., 2021). The MI-related targets were found by searching these databases with the term “Myocardial Infarction”. The potential therapeutic targets for treating MI were obtained from the GeneCards, MalaCards, DisGeNET, TTD and GEO databases, and were filtered to include targets that appeared in at least two of these databases. Subsequently, these targets were cross-referenced with the targets of XLGB treatment to identify potential targets for XLGB in the treatment of MI.

### 2.8 Network analysis

We imported the potential targets into the STRING 12.0 database (https://string-db.org/) to obtain the protein-protein interaction (PPI) network, removed isolated nodes, and then visualized the results using Cytoscape 3.10.0. In addition, the gene ontology (GO) of the potential target set for XLGB treatment of MI was analyzed by Metscape (http://metascape.org/gp/#/main/step1) (Zhou et al., 2019). R software package and clusterProfiler (version 3.14.3) were utilized to analyze Kyoto Encyclopedia of Genes and Genomes (KEGG) pathway. Set minimum gene set to 5, maximum gene set to 5000, *p*-values were adjusted using a BH approach, statistical significance was denoted if adjust *p*-value <0.05, false discovery rate (FDR) < 0.1 (Zeng et al., 2021).

### 2.9 Molecular docking

Based on the results of network pharmacology analysis, to further validate and assess the effective binding of XLGB active ingredients to their predicted targets, XLGB’s main active ingredients, including icariin (ICA), icariside II (ICS II), mangiferin, bavachin, corylifol A, tanshinoneⅡ, neobavaisoflavone, rehmannioside D, isobavachin and psoralen were separately docked with three targets (NLRP3, Caspase3 and RIP1) in the PANoptosis pathway. Briefly, PubChem database was utilized to collect 3D molecular structures of XLGB’s main active ingredients, and RCSB Protein Data Bank (PDB, http://www.RCSB.org/) was used to obtain structural files for the target proteins. PyMOL 4.6.0 was employed to remove non-crystalline water molecules from the structures of NLRP3 (PDB ID: 6npy), Caspase3 (PDB ID: 6ckz), and RIP1 (PDB ID: 4*i*th). Subsequently, molecular structures and targets were uploaded to DockThor (https://dockthor.lncc.br/v2/) for docking, and the results were downloaded (Santos et al., 2020). The ingredients with the highest binding affinity for NLRP3, Caspase3, and RIP1 were visualized using UCSF ChimeraX 1.6.1 (Pettersen et al., 2021).

### 2.10 Molecular dynamic (MD) simulation

As described in our previous studies, and based on the results of molecular docking, the complexes with the highest binding affinity (Caspase3 and ICA; NLRP3 and ICS II; RIP1 and corylifol A) were selected for MD simulation using GROMACS(Sellis et al., 2009). In brief, the CGenFF program (https://cgenff.silcsbio.com/) was used to generate the topology file for the ligand, which was then combined with the topology file of the protein to form the complex (Vanommeslaeghe and MacKerell, 2012). After energy minimization and equilibration, molecular dynamics simulations were initiated using the CHARMM36 force field (Ruda et al., 2023). The simulation process consisted of 5,000,000 steps with a total duration of 100 nanoseconds (ns). After completing the simulation, trajectory data were subjected to periodic corrections. Root Mean Square Deviation (RMSD) and Root Mean Square Fluctuation (RMSF) data for each amino acid in the trajectory were extracted and curve graphs were generated.

### 2.11 TUNEL staining assay

To assess myocardial cell death, tissue sections were subjected to TUNEL staining according to the instructions provided in the TUNEL Apoptosis Assay Kit. Briefly, tissue sections (4 μm) were incubated in TUNEL reaction solution for 60 min. Subsequently, antifade mounting medium with DAPI was applied to mount the slides. Apoptotic cells were observed under a fluorescence microscope (BX 53 Olympus, Japan), and the number of apoptotic cells was quantified using ImageJ software (https://ij.imjoy.io/) was used to calculate the apoptotic cell rate.

### 2.12 Immunofluorescence (IF) staining

IF staining was used to ascertain the location of PANoptosis-related proteins. In brief, tissue sections (4 μm) were deparaffinized using a Leica Autostainer XL (ST5010), and then were treated with an immunostaining fixation solution for 6 min to prevent section detachment. Subsequently, the sections were blocked with an immunostaining blocking solution. The sections were then incubated overnight at 4°C with primary antibodies, including NLRP3 (1:50, Proteintech), Cleaved-caspase3 (1:100), p-MLKL (1:100), CoraLite 488 (1:500), and CoraLite 594 (1:500). Subsequent to primary antibody incubation, the sections were incubated with the corresponding secondary antibodies for 4 h. The slides were mounted with antifade mounting medium with DAPI and observed using a laser confocal microscope (Leica STELLARIS 5, Germany). The ImageJ software was utilized for the three-dimensional visualization and quantification of fluorescence intensity (https://ij.imjoy.io/).

### 2.13 ELISA

To detect myocardial damage as well as OS and inflammatory factors, serum levels of these were measured using an ELISA kit. Assays for CK-MB, cTn-I, cTnT, AST, IL-1β, IL-1α, TNF-α, IL-6, ROS, MDA, CAT, SOD, GSH, and GSH-Px were performed using standard ELISA kits following the manufacturer’s instructions.

### 2.14 Western blot

Tissue assessment was carried out using the BCA protein assay as described in our previous study (Wu et al., 2023). Subsequently, the lysates were normalized to equal amounts per group, and 10 μg of protein from the tissue lysates were separated using 12% sodium dodecyl sulfate-polyacrylamide gel electrophoresis. The separated proteins were then transferred to polyvinylidene fluoride membranes, which were blocked with a 1% blocking buffer for 30 min at room temperature. After blocking, the membranes were incubated with primary antibodies, including NLRP3 (1:1,000), GSDMD-N (1:1,000), Caspase1 (1:1,000), Cleaved-caspase1, p20 (1:1,000), Caspase3 (1:2000), Cleaved-caspase3 (1:2000), RIP1 (1:1,000), p-RIP1 (1:1,000), RIP3 (1:1,000), p-RIP3 (1:1,000), MLKL (1:1,000), and p-MLKL (1:1,000) overnight at 4°C. Following incubation, proteins were detected using species-specific HRP-conjugated secondary antibodies for 1 h at room temperature. Representative bands were visualized using ECL WB reagents, and the relative optical band intensities were quantified using ImageJ software (https://ij.imjoy.io/).

### 2.15 Statistics

The data are presented as the mean ± standard deviation (SD) of 5 or 6 independent experiments and were analyzed using SPSS version 29.0 (SPSS, Inc., Chicago, USA). Statistical comparisons among three or more groups were conducted using one-way ANOVA, followed by the Bonferroni *post hoc* test (assuming equal variances). A significance level of *p* < 0.05 was considered statistically significant.

## 3 Result

### 3.1 XLGB mitigated MI-induced myocardial injury in mice

**FIGURE 1:**
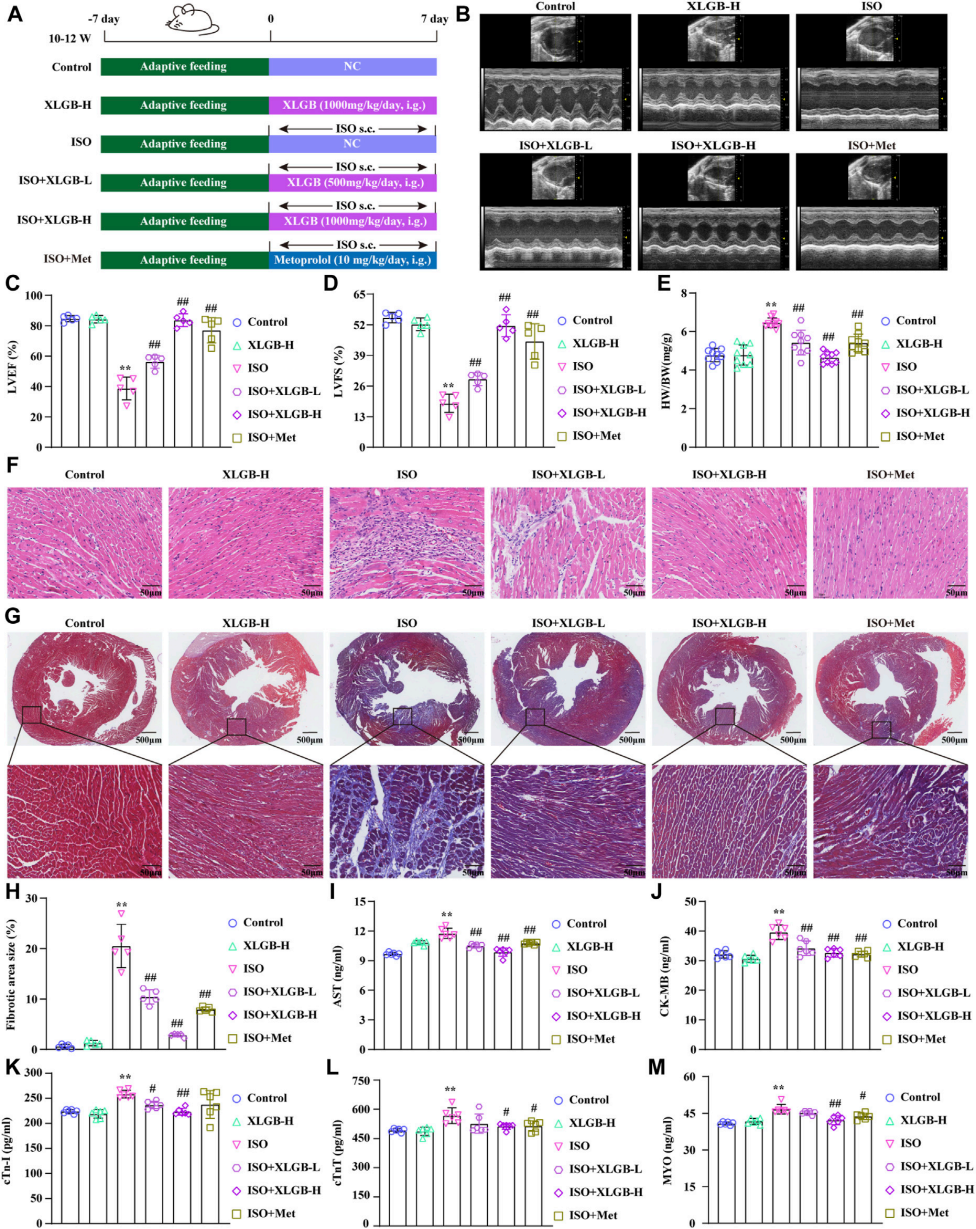
XLGB significantly ameliorated ISO-induced myocardial injury. **(A)** Schematic presentation of the study protocol. **(B)** Echocardiographic representation. **(C)** LVEF (n = 5). **(D)** LVFS (n = 5). **(E)** Cardiac organ coefficients (n = 9). **(F)** HE (×40, scale bar: 50 μm). **(G)** Masson staining (×4, scale bar: 500 μm). **(H)** Collagen volume fraction (n = 5). **(I)** AST (n = 6). **(J)** CK-MB (n = 6). **(K)** cTn-I (n = 6). **(L)** cTnT (n = 6). **(M)** MYO (n = 6). Values are expressed as the mean ± SD. ^**^ *p* < 0.01 versus Control group. ^##^ *p* < 0.01 versus ISO group.

We used ISO-induced MI mouse model to investigate the impact of XLGB on myocardial injury (Figure 1A). The results demonstrated that ISO increased ejection fraction (EF) and fractional shortening (FS) (Figures 1B–D) and induced structural and functional abnormalities in the heart, as indicated by an increased heart weight-to-body weight ratio (HW/BW) (Figure 1E). However, treatment with XLGB (500 and 1,000 mg/kg/day) significantly reversed these conditions, improving both the cardiac structure and function. Subsequently, the effects of XLGB on pathological changes in mouse myocardial tissue were observed through HE staining and Masson staining (Figures 1F–H). The results showed that compared with the control group, mice in the ISO group showed significant cardiac hypertrophy and inflammatory infiltration. XLGB (500 and 1,000 mg/kg/day) significantly reduced these symptoms, and the degree of myocardial fibrosis was also reduced. Furthermore, compared with the ISO group, XLGB (500 mg/kg) significantly inhibited AST, CK-MB and cTn-I. However, XLGB (1000 mg/kg) significantly reduced the levels of AST, CK-MB, cTn-I, cTnT and MYO (Figures 1I–M). Interestingly, XLGB (500 mg/kg/day) can only alleviate the myocardial damage caused by ISO to a certain extent, but the effect of XLGB (1000 mg/kg/day) is better than that of the positive drug group. These findings suggest that XLGB effectively alleviates ISO-induced myocardial injury in mice.

### 3.2 Targets for XLGB and MI

**FIGURE 2:**
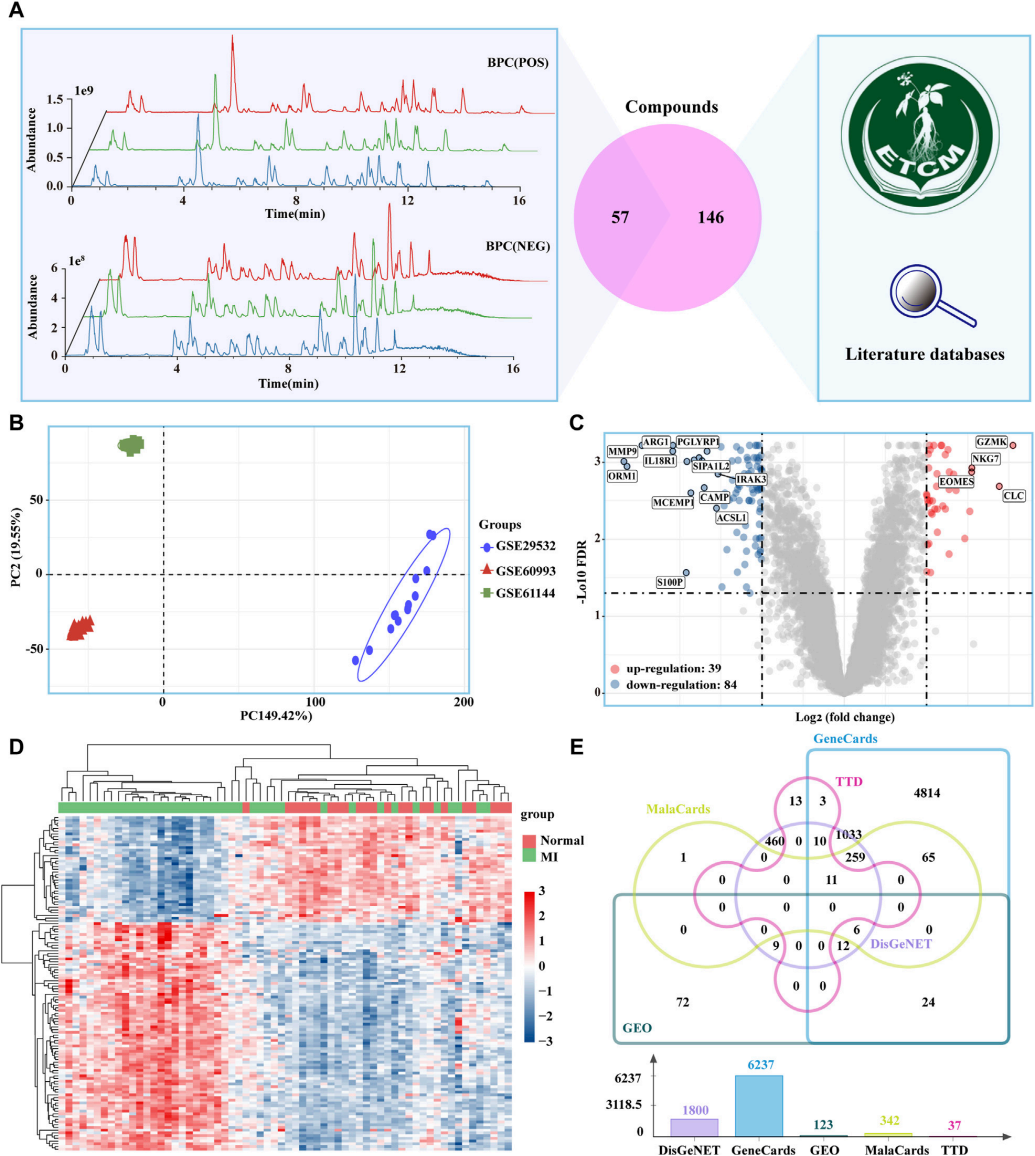
Potential targets of XLGB in the treatment of MI. **(A)** 57 Main ingredients from XLGBA were identified by UPLC-QE-Orbitrap-HRMS; 146 ingredients contained in XLGB were collected from databases and the literature. **(B)** PCA. **(C)** MI-related DEGs obtained from the GEO database **(D)** Heatmap of DEGs. **(E)** Therapeutic targets of MI.

A total of 900 ingredients were identified using UPLC-QE-Orbitrap-HRMS. The ingredients with relatively high content in the XLGB include ilexoside II, Epmedin C, Neobavaisoflavone, and Icariin (Supplementary Figure S1). These ingredients were predominantly classified into the following categories: flavonoids (47.73%), terpenoids (16.37%), phenolic compounds (13.05%), steroids (8.11%), and carbohydrates and glycosides (6.55%) (Supplementary Figure S2). We obtained 57 active ingredients and identified 676 potential target genes (Supplementary Table S1). 146 ingredients contained in XLGB were collected from, ETCM databases and the literature (Figure 2A). Together, these ingredients act on 1,037 potential targets. The PCA results indicate significant differences among the three gene sets (Figure 2B). In addition, 123 DEGs were screened out through the GEO database (Figures 2C, D). The potential therapeutic targets for treating MI were obtained from the GeneCards, MalaCards, DisGeNET, TTD, and GEO databases. A total of 1,432 targets were selected based on their presence in at least two of these databases (Figure 2E).

### 3.3 Potential pharmacological mechanisms of XLGB in the treatment of MI

**FIGURE 3:**
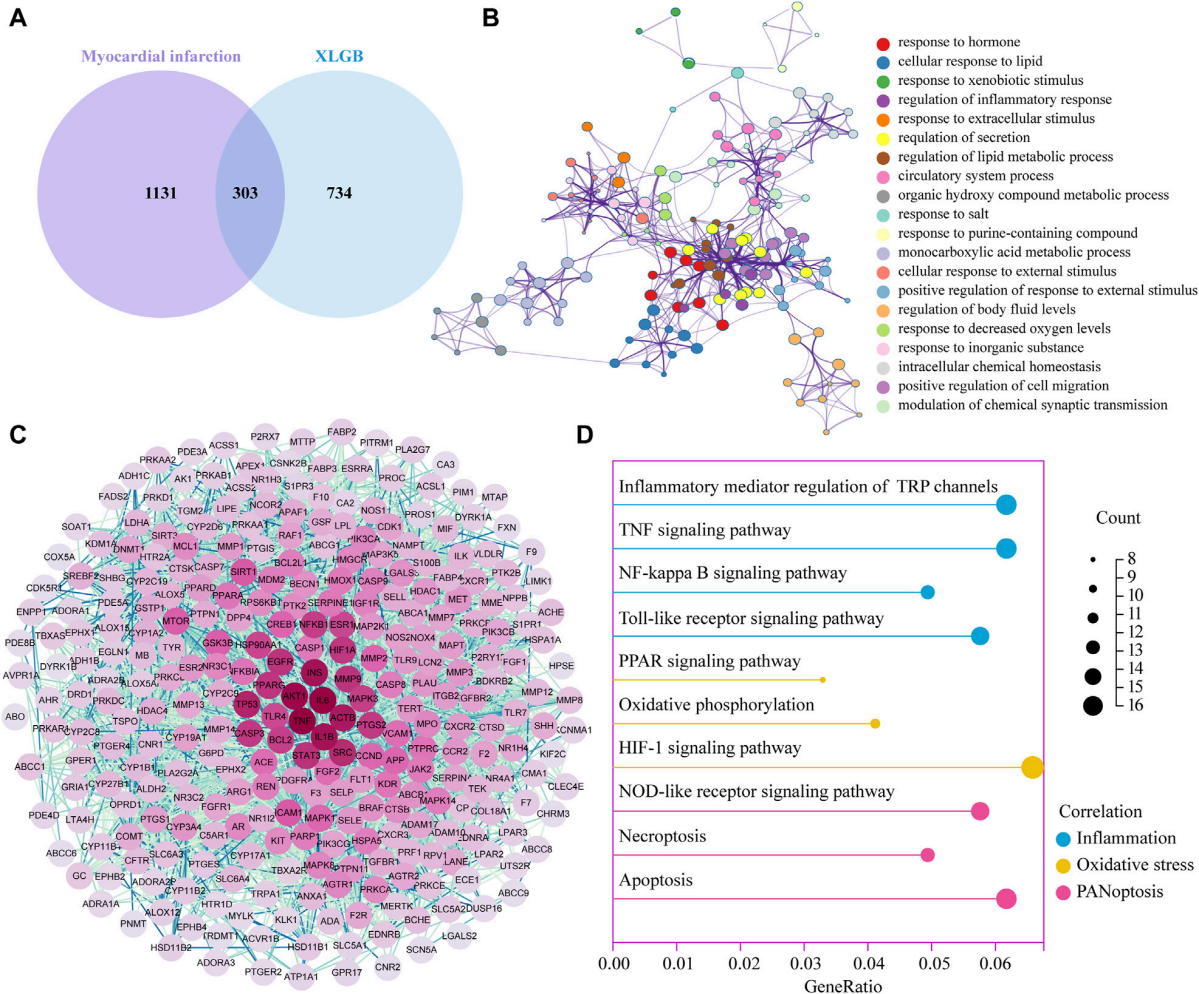
Potential pharmacological mechanisms of XLGB in the treatment of MI. **(A)** The XLGB target intersected with MI target. **(B)** GO function enrichment analysis. **(C)** Diagram of the core target protein interaction network. **(D)** KEGG pathway enrichment analysis.

Subsequently, a Venn analysis revealed that 303 potential targets for XLGB in the treatment of MI (Figure 3A). GO analysis of these potential targets indicated that XLGB treatment of MI is associated with the regulation of processes such as response to xenobiotic stimulus, regulation of inflammation response and response to decreased oxygen levels (Figure 3B). The potential targets were further analyzed in the String database to investigate PPI. We ranked the targets based on their degree values, with darker node colors indicating higher degrees and a stronger correlation with MI. The results revealed that the core target genes in the PPI network mainly included IL-6, TNF-α, IL-1β, and Caspase3, among others (Figure 3C). KEGG enrichment analysis confirmed that the protective effect of XLGB on MI is associated with OS, inflammatory responses, and the PANoptosis signaling pathway, which includes apoptosis, necroptosis, and pyroptosis (Figure 3D).

### 3.4 XLGB bioactive ingredients directly bound to key targets of PANoptosis

**FIGURE 4:**
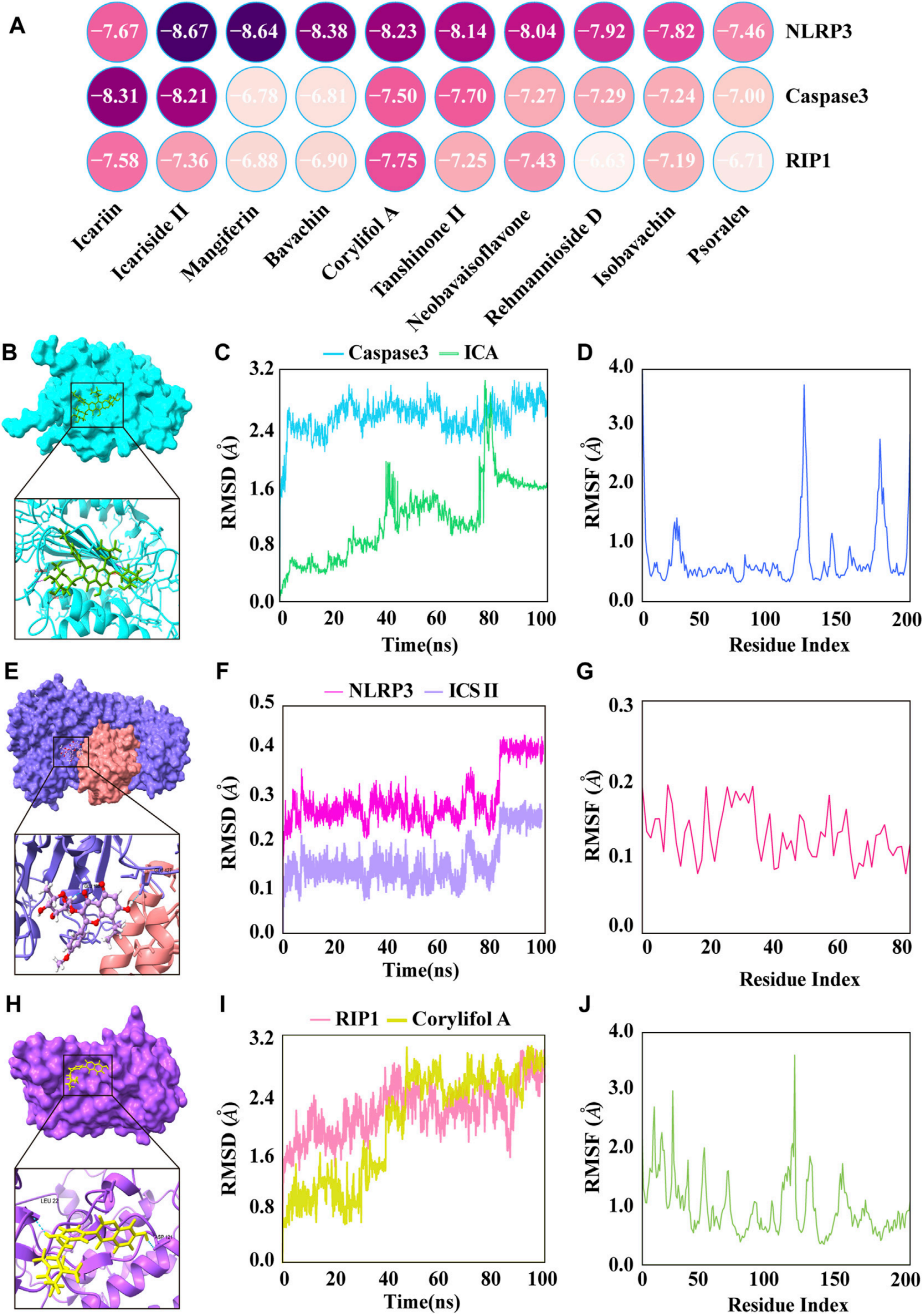
The major active ingredients of XLGB bound directly to key targets of PANoptosis. **(A)** Heatmap of molecular docking. **(B)** ICA bound to Caspase3. **(C)** The RMSD of the ICA-Caspase3 complex. **(D)** RMSF of residues of the whole protein in the ICA-Caspase3 complex. **(E)** ICS II bound to NLRP3. **(F)** The RMSD of the ICS II-NLRP3 complex. **(G)** RMSF of residues of the whole protein in the ICS II-NLRP3 complex. **(H)** Corylifol A bound to RIP1. **(I)** The RMSD of the Corylifol A-RIP1 complex. **(J)** RMSF of residues of the whole protein in the Corylifol A-RIP1 complex.

To further validate and assess the effective binding of XLGB bioactive ingredients to their predicted targets, main active ingredients were individually docked with three targets in the PANoptosis pathway (NLRP3, Caspase3, and RIP1) based on network pharmacology results. The binding energies for all ligands were greater than −5 kcal/mol, indicating strong binding affinities between all ligands and their respective receptors (Figure 4A). Among them, the ingredient with the strongest binding to the target Caspase3 was ICA with a binding energy of −8.31 kcal/mol (Figure 4B). Furthermore, the RMSD indicates the structural stability during the 100 ns MD simulation. The results showed that RMSD reached a plateau after 80 ns (Figure 4C). RMSF has a large fluctuation range, which means greater flexibility of amino acid residues in the protein, indicating possible structural changes in the protein (Figure 4D). The ingredients with the strongest binding to the target NLRP3 was ICS II with a binding energy of −8.67 kcal/mol (Figure 4E). The RMSD demonstrating that the protein structure remains stable throughout the simulation (Figure 4F). RMSF values below 0.2 Å revealed that these residues exhibit greater rigidity due to their interaction with ICS II (Figure 4G). The ingredients with the strongest binding to the target RIP1 was corylifol A with a binding energy of −7.75 kcal/mol (Figure 4H). The results showed that RMSD reached equilibrium at 90ns and stabilized at about 3 Å (Figure 4I). The RMSF value fluctuates within a certain range, which means that the flexibility of amino acid residues in the protein is general and the complex structure is stable (Figure 4J).

### 3.5 XLGB reduced myocardial apoptosis and p-MLKL, NLRP3, Cleaved-caspase3 expression

**FIGURE 5:**
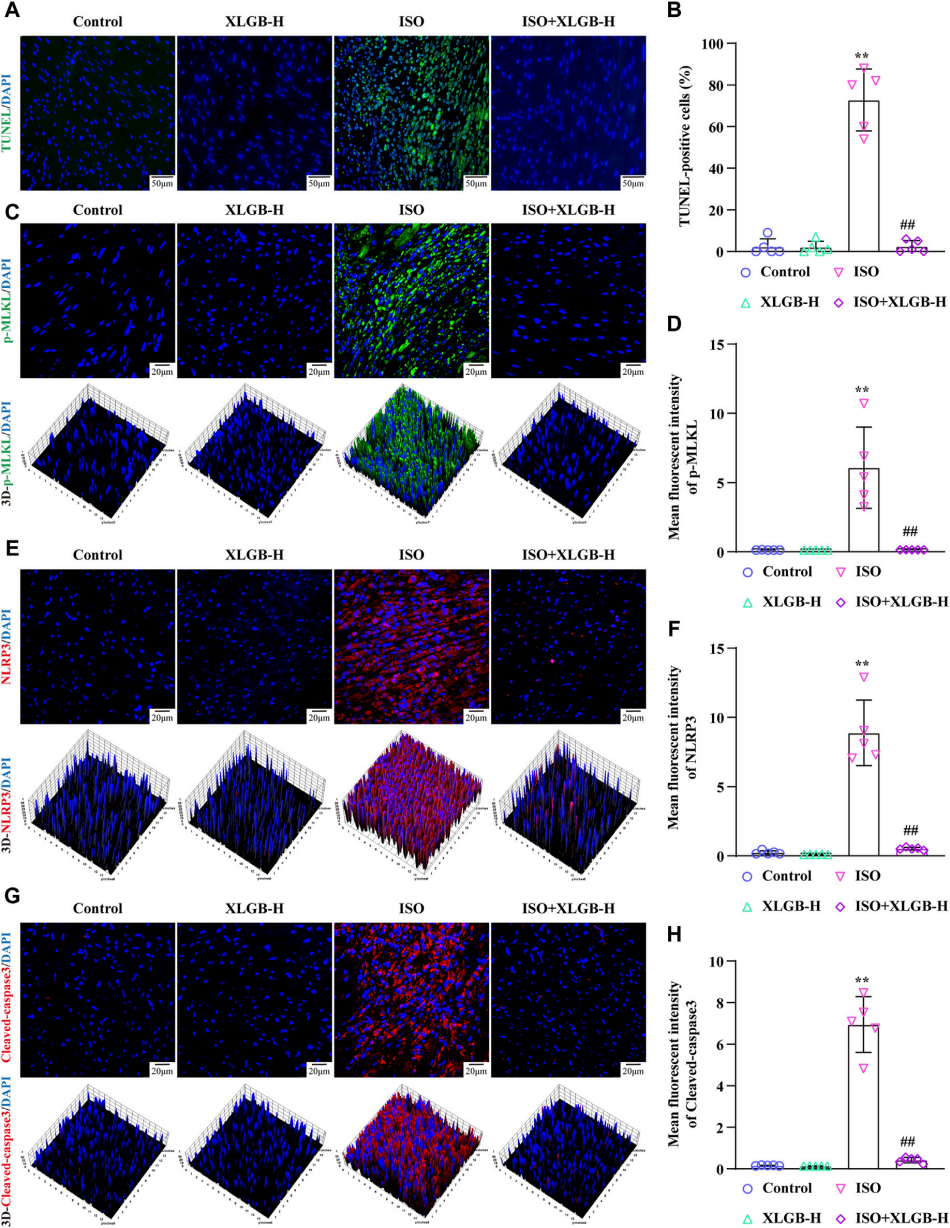
XLGB protected against MI-induced myocardial apoptosis by reducing p-MLKL, NLRP3, Cleaved-caspase3 protein expressions. **(A)** Representative images of TUNEL staining (×40; scale bar: 50 μm). **(B)** Quantitation of TUNEL positive cells (n = 5). **(C)** Representative images of p-MLKL expression (×63; scale bar: 20 μm). **(D)** p-MLKL intensity (n = 5). **(E)** Representative images of NLPR3 protein expression (×63; scale bar: 20 μm). **(F)** NLRP3 intensity (n = 5). **(G)** Representative images of Cleaved-caspase3 protein expression (×63; scale bar: 20 μm). **(H)** Cleaved-caspase3 intensity (n = 5). Values are expressed as the mean ± SD. ^**^ *p* < 0.01 versus Control group. ^##^ *p* < 0.01 versus ISO group.

Next, we investigated the impact of XLGB on MI-induced cell PANoptosis. The results demonstrated that XLGB reduced myocardial cell PANoptosis induced by MI, as evidenced by a decrease in the number of TUNEL-positive cell nuclei (Figures 5A, B). Furthermore, to further corroborate the results from network pharmacology analysis, we conducted immunofluorescence experiments to assess the expression of p-MLKL, NLRP3, and Cleaved-caspase3 in myocardial cells. The findings revealed that a significant increase in the expression of p-MLKL, NLRP3, and Cleaved-caspase3 induced by MI, while treatment with XLGB markedly reduced their expression (Figures 5C–H). These findings suggest that XLGB can effectively protect against myocardial cell apoptosis by inhibiting key proteins in the PANoptosis pathway, including p-MLKL, NLRP3, and Cleaved-caspase3.

### 3.6 XLGB inhibited myocardial injury through downregulated PANoptosis signaling pathway after MI in mice

**FIGURE 6:**
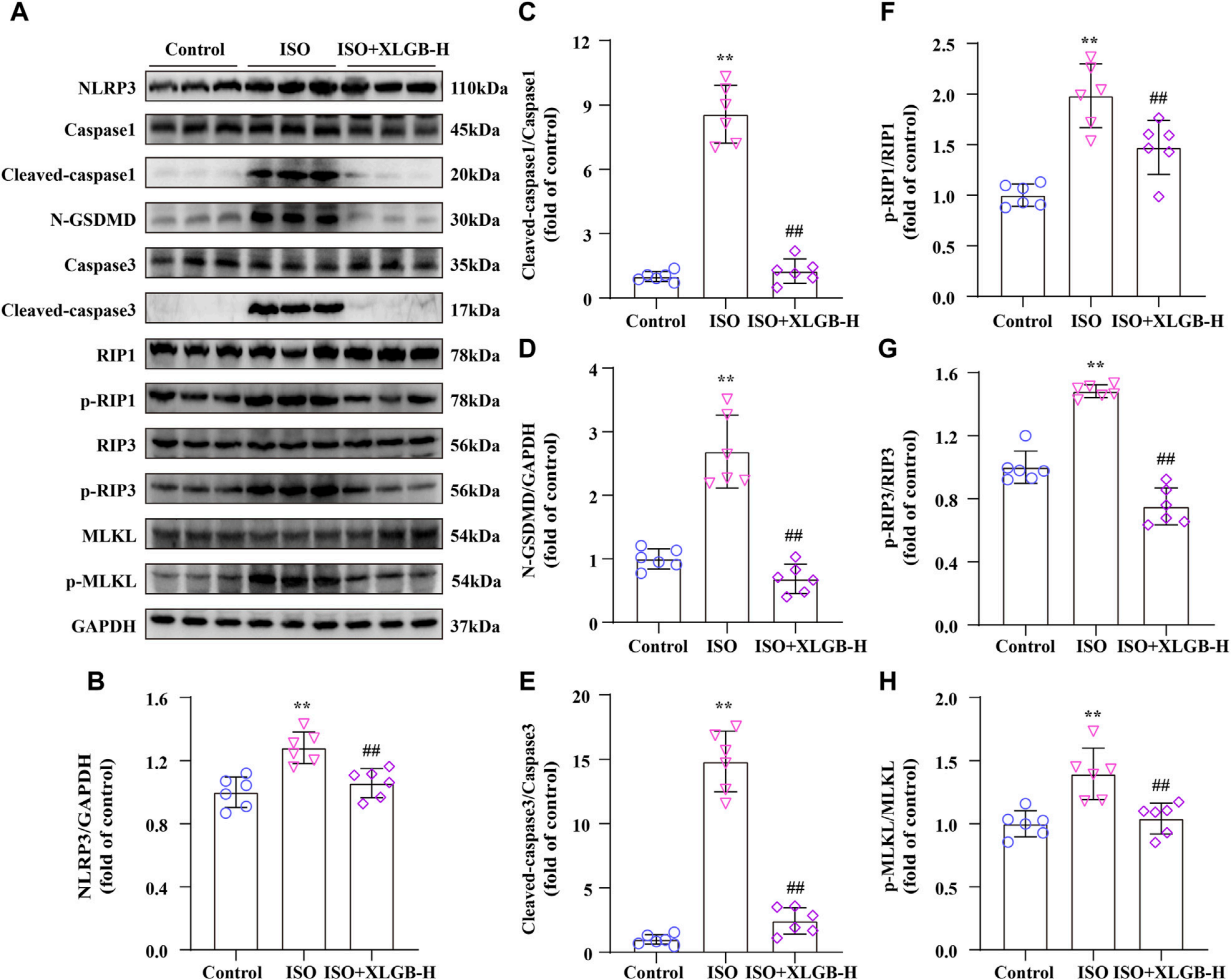
XLGB inhibited myocardial injury through downregulated PANoptosis signaling pathway after MI in mice. **(A)** Representative western blots of PANoptosis-related proteins. **(B–H)** Western blot results were quantified (n = 6). Values are expressed as the mean ± SD. ^**^ *p* < 0.01 versus Control group. ^##^ *p* < 0.01 versus ISO group.

In order to investigate the relationship between XLGB and PANoptosis in mice with ISO-induced MI, we analyzed the expression of PANoptosis-related proteins using western blots (Figure 6A). The results showed that following ISO treatment, markers of pyroptosis (NLRP3/Cleaved-caspase1/N-GSDMD) (Figures 6B–D), apoptosis (Cleaved-caspase3) (Figure 6E) and necroptosis (p-RIP1/p-RIP3/p-MLKL) (Figures 6F–H) were significantly upregulated. However, these changes were significantly reversed by XLGB. Overall, we infer that XLGB is involved in regulating ISO-induced cardiomyocyte PANoptosis.

### 3.7 XLGB decreased proinflammatory cytokines and OS mediators

**FIGURE 7:**
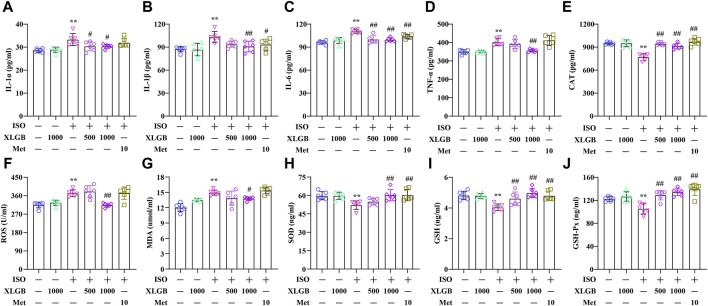
XLGB decreased proinflammatory cytokines and OS mediators. **(A)** IL-1α level (n = 6). **(B)** IL-1β level (n = 6). **(C)** IL-6 level (n = 6). **(D)** TNF-α level (n = 6). **(E)** CAT level (n = 6). **(F)** ROS level (n = 6). **(G)** MDA level (n = 6). **(H)** SOD level (n = 6). **(I)** GSH level (n = 6). **(J)** GSH-Px level (n = 6). Values are expressed as the mean ± SD. ^**^ *p* < 0.01 versus Control group. ^##^ *p* < 0.01 versus ISO group.

XLGB treatment significantly suppressed the expression levels of inflammatory cytokines, demonstrating robust anti-inflammatory activity. The results showed that MI led to a substantial increase in various inflammatory cytokines, including IL-1α, IL-1β, IL-6, and TNF-α. In contrast, XLGB treatment markedly reduced the levels of these inflammatory cytokines (Figures 7A–D). OS, akin to inflammation, represented a significant pathological process accompanying MI. Building on the outcomes of our previous network analysis, ELISA assays were employed to quantify OS mediators. In the mouse model of MI, there were elevated levels of cellular and mitochondrial ROS as well as MDA, along with decreased CAT, SOD, GSH, and GSH-Px activities when compared to the control group. However, these alterations were reversed by XLGB treatment following the MI insult (Figures 7E–J). These findings strongly suggest that XLGB effectively inhibits the development of MI-induced inflammation and OS.

## 4 Discussion

**FIGURE 8:**
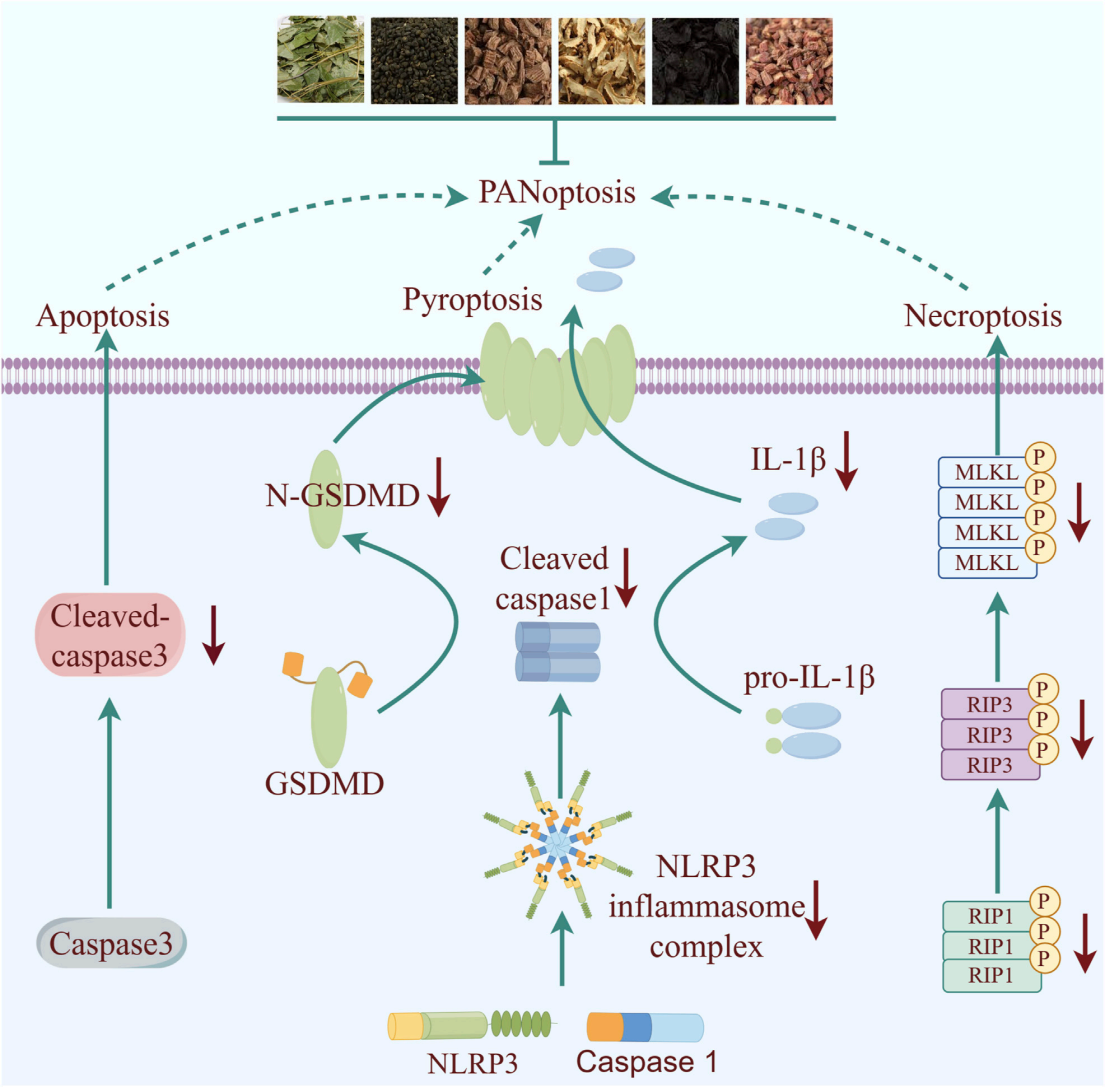
Schematic illustration of molecular mechanisms for the protective effect of XLGB against MI. XLGB evokes robust protection on MI through PANoptosis.

This study reveals for the first time that XLGB exerts cardioprotective effects due to mitigate inflammation and OS through mediating the PANoptosis signaling pathway (Figure 8). Our results collectively suggest potential therapeutic targets for XLGB in the treatment of MI and broaden the scope of potential clinical applications for XLGB.

Early intervention is crucial for reducing myocardial damage in the treatment of MI. However, challenges in delivering timely medical care to patients have presented an ongoing obstacle in the search for more effective treatment methods. Research has shown that inflammation and OS are significant factors in worsening myocardial damage following MI (Li H. et al., 2022). Hence, it is imperative for drugs aimed at treating MI to exhibit strong anti-inflammatory and antioxidant properties. Prior studies have demonstrated the efficacy of *Epimedium brevicornu* Maxim. along with its primary active metabolites ICS II and ICA, in combating a range of diseases owing to their potent anti-inflammatory and antioxidant attributes (Liu et al., 2020; Wu et al., 2023). Consequently, the pharmacological profile of XLGB has motivated further investigation into its therapeutic potential for MI and its associated molecular targets. The results of our study demonstrate that XLGB effectively mitigated myocardial fibrosis and inflammation infiltration, leading to the restoration of cardiac ejection function. ELISA analysis revealed that XLGB significantly suppressed levels of AST, CK-MB, and cTn-I. It is noteworthy that XLGB had a modest impact on cTnT and MYO, potentially attributable to the suboptimal dosage of 500 mg/kg. These findings support the potential of XLGB as a promising therapeutic intervention for ischemic heart disease, offering protective benefits. Further in-depth research is necessary to elucidate the precise mechanism of action. Therefore, network pharmacology and molecular docking simulations were utilized to predict the potential underlying mechanisms of XLGB in MI. The findings suggest that OS and PANoptosis-related pathways play a role in the beneficial effects of XLGB post-MI. Interestingly, the primary active ingredients of XLGB, such as ICA, ICS II, mangiferin, bavachin, corylifol A, tanshinoneⅡ, neobavaisoflavone, rehmannioside D, isobavachin, psoralen, can directly interact with NLRP3, Caspase3, and RIP1. The results indicate that XLGB may play a role in mitigating the pathological advancement of MI through modulation of the NLRP3/Caspase3/RIP1 pathway. Numerous studies have demonstrated the activation of NLRP3-mediated pyroptosis, Cleaved-caspase3-mediated apoptosis, and p-MLKL-mediated necroptosis shortly after MI, leading to myocardial inflammation and cell death. The results demonstrate that XLGB effectively decreased the expression levels of NLRP3, Cleaved-caspase3, and p-MLKL in myocardial cells, leading to a reduction in myocardial cell death as indicated by IF and TUNEL staining. Additionally, in line with the predictions from network pharmacology analysis, XLGB also suppressed the protein levels of PANoptosis-related genes post-MI. These findings suggest that XLGB mitigates MI-induced myocardial damage by inhibiting the PANoptosis signaling pathway, while also decreasing levels of pro-inflammatory cytokines and OS. Nonetheless, our study presents empirical evidence supporting the potential of XLGB in ameliorating ISO-induced myocardial injury. However, the specific mode (apoptosis, necrosis, or pyroptosis) through which XLGB exerts its beneficial effects on MI remains unclear. Additionally, the pharmacokinetic profile, tissue distribution, and hepatic metabolism of XLGB warrant further investigation. The main active ingredients of XLGB are yet to be further experimentally verified, which can be explored in subsequent studies by surface plasmon resonance experiments, microscale thermophoresis method, isothermal titration calorimetry and other methods for exploration. These pending issues will be elucidated and ultimately by well-designed and large cohort clinical trials in our following story.

In conclusion, this study discloses that XLGB exerts potent protective effects against MI by inhibiting myocardial cell PANoptosis. Therefore, our findings highlight XLGB may be a promising weapon in combating MI.

## Data Availability

The original contributions presented in the study are included in the article/Supplementary Material, further inquiries can be directed to the corresponding authors.
