# Supplementary material for: Targeting the PANoptosis signaling pathway for myocardial protection: therapeutic potential of Xian Ling Gu Bao capsule
Source: Front Pharmacol. 2024 May 10;15:1391511. doi: 10.3389/fphar.2024.1391511 (PMC11116727; doi:10.3389/fphar.2024.1391511)
Supplement: Supplementary file 3 [file DataSheet1.docx]

Supplementary Material

## Supplementary Figures


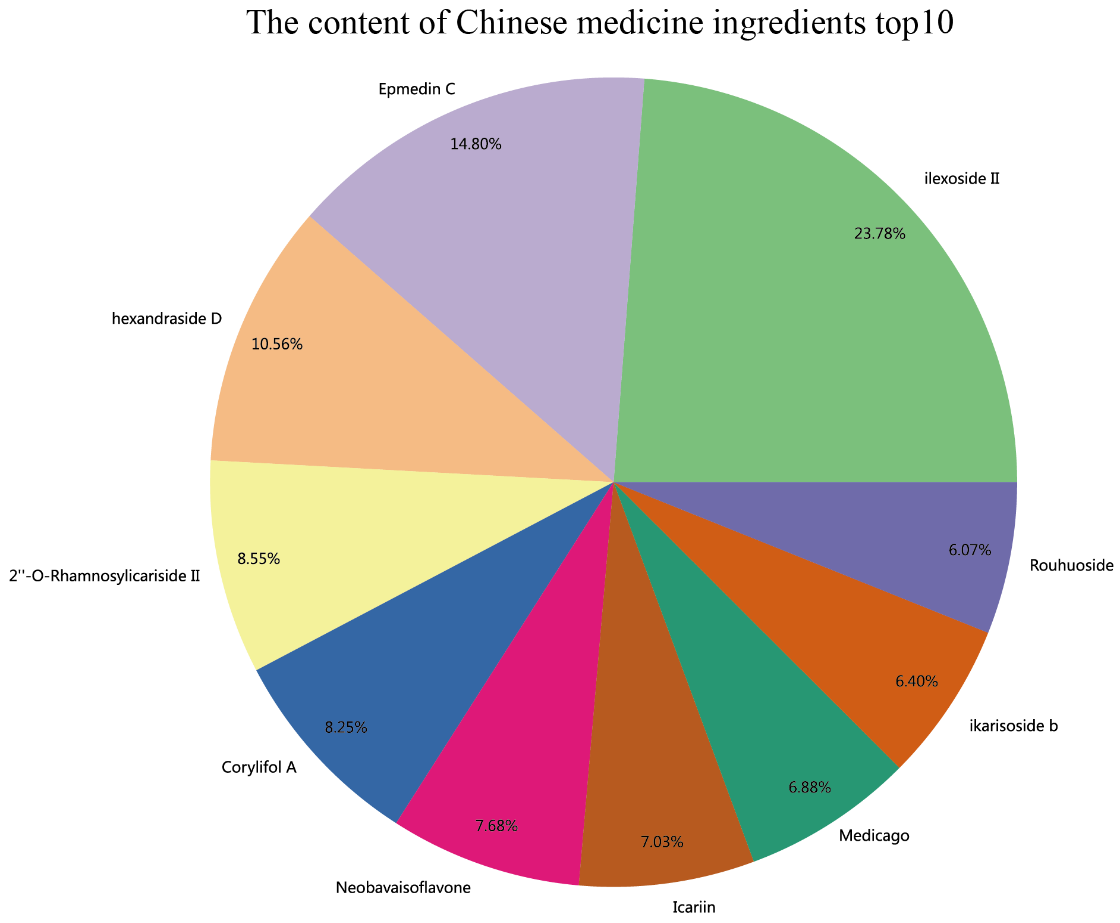


**Supplementary** **Figure S1.** The content of Chinese medicine ingredients top10


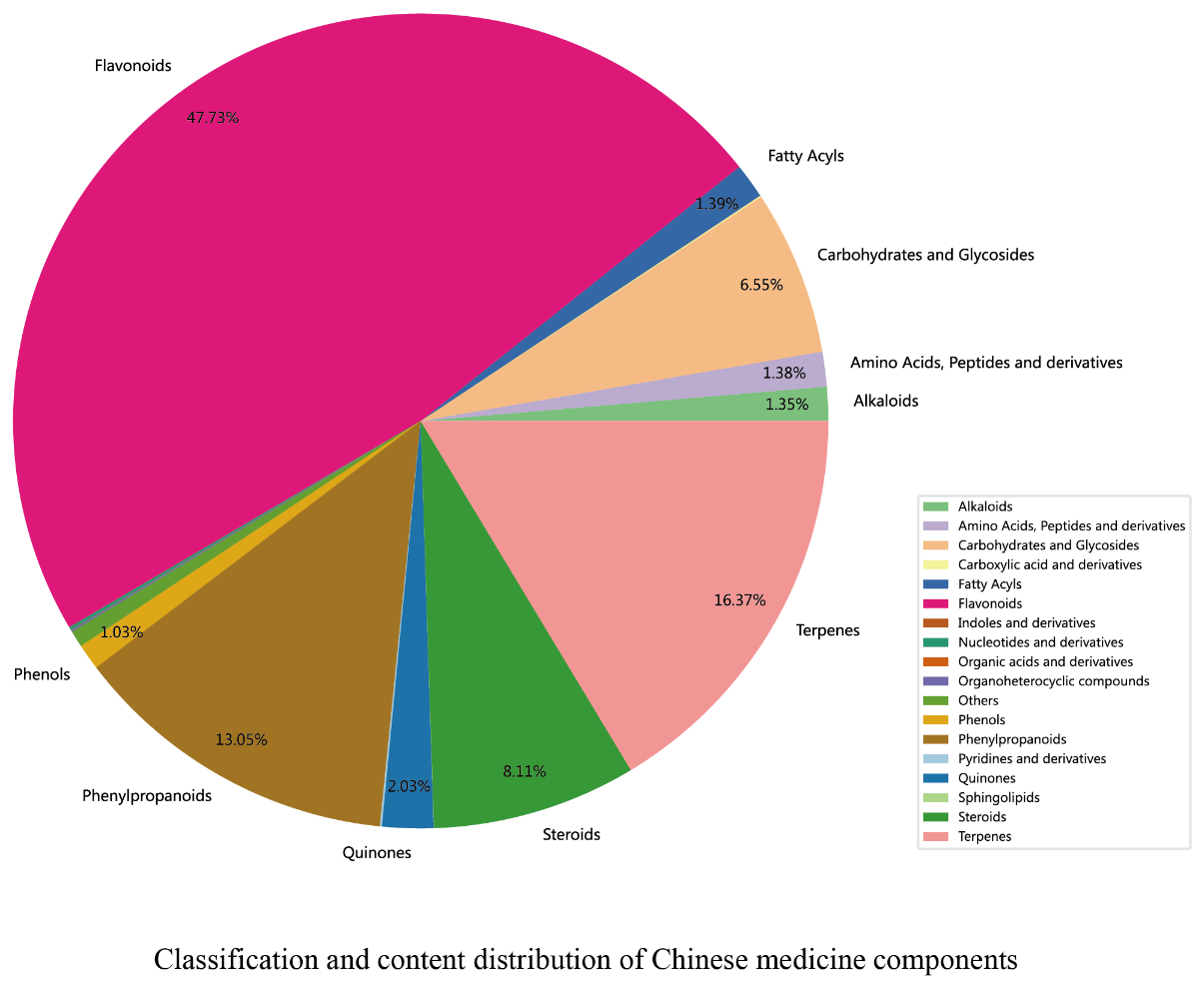


**Supplementary Figure S2.** Classification and content distribution of Chinese medicine components
