# Supplementary material for: Targeting the PANoptosis signaling pathway for myocardial protection: therapeutic potential of Xian Ling Gu Bao capsule
Source: Front Pharmacol. 2024 May 10;15:1391511. doi: 10.3389/fphar.2024.1391511 (PMC11116727; doi:10.3389/fphar.2024.1391511)

Raw data of western blot

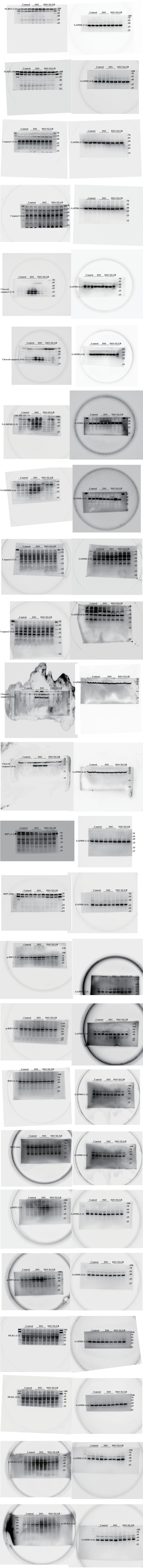

Supplement: Supplementary file 1 [file DataSheet2.pdf]
